# Improve quality of patient care for systemic lupus erythematosus in China by enhancing the construction of Centers of Excellence

**DOI:** 10.2478/rir-2023-0023

**Published:** 2023-09-27

**Authors:** Mengtao Li, Xinping Tian, Qian Wang, Jiuliang Zhao, Junyan Qian, Chengde Yang, Cibo Huang, Hui Luo, Huji Xu, Jieruo Gu, Jin Lin, Lijun Wu, Miaojia Zhang, Niansheng Yang, Pinting Yang, Shengyun Liu, Wei Wei, Xinwang Duan, Yi Liu, Zhiyi Zhang, Zhuoli Zhang, Yan Zhao, Xiaofeng Zeng

**Affiliations:** Department of Rheumatology, Peking Union Medical College Hospital, National Clinical Research Center for Dermatologic and Immunologic Diseases, Beijing, China; Department of Rheumatology, Ruijin Hospital, Shanghai Jiao Tong University School of Medicine, Shanghai, China; Department of Rheumatology and Immunology, Shenzhen University Affiliated South China Hospital, Shenzhen, Guangdong Province, China; Department of Rheumatology, Xiangya Hospital of Central South University, Changsha, Hunan Province, China; Department of Rheumatology and Immunology, Shanghai Changzheng Hospital, Naval Medical University, Shanghai, China; Department of Rheumatology and Immunology, The Third Affiliated Hospital of Sun Yat-sen University, Guangzhou, Guangdong Province, China; Department of Rheumatology, The First Affiliated Hospital, Zhejiang University School of Medicine, Hangzhou, Zhejiang Province, China; Department of Rheumatology, Xinjiang Uygur Autonomous Region People’s Hospital, Urumqi, Xinjiang Uygur Autonomous Region, China; Department of Rheumatology, Jiangsu Province Hospital, Nanjing, Jiangsu Province, China; Department of Rheumatology and Immunology, The First Affiliated Hospital of Sun Yat-sen University, Guangzhou, Guangdong Province, China; Department of Rheumatology, The First Hospital of China Medical University, Shenyang, Liaoning province, China; Department of Rheumatology, The First Affiliated Hospital of Zhengzhou University, Zhengzhou, Henan Province, China; Department of Rheumatology, Tianjin Medical University General Hospital, Tianjin, China; Department of Rheumatology, The Second Affiliated Hospital of Nanchang University, Nanchang, Jiangxi Province, China; Department of Rheumatology and Immunology, West China Hospital, Sichuan University, Chengdu, Sichuan Province, China; Department of Rheumatology, the First Affiliated Hospital of Harbin Medical University, Harbin, Heilongjiang Province, China; Department of Rheumatology, Peking University First Hospital, Beijing, China

Systemic lupus erythematosus (SLE) is an autoimmune disease characterized by diverse clinical manifestations and multi-organ involvement.^[1]^ The management of this difficult and complicate disease is still a big challenge, even in countries with advanced health care resources. China has a large patient population with SLE. It is estimated that at least 1 million patients with SLE in mainland China, this number is still increasing as the diagnostic level is significantly improved. China has the highest disease burden of SLE in the entire world.^[2,3]^ Therefore, establishing concrete and applicable strategy is crucial to manage such a huge patient population with limited number of rheumatologists in China. Under the leadership of National Clinical Center for Dermatologic and Immunologic Center (NCRC-DID) of China, a series of strategies have been established to prompt standardized treatment and management of SLE across the country. Hereby we report one of the approaches, developing centers of excellence (COEs) for SLE and their management.

The specialty of Rheumatology was established in 1970s in China. The disparity in development among regions and proficiency level among healthcare providers is significant across the country. ^[2]^ In recent years, improving the quality of care for patients with rheumatic diseases has become a high priority of National Health Commission of the People’s Republic of China. The Guidelines for the Construction and Management of Departments of Rheumatology in General Hospitals and the Guidelines for the Basic Standards of Department of Rheumatology in General Hospitals were released in 2019 by the National Health Commission of the People’s Republic of China. Furthermore, the 2020 Chinese Guidelines for the Diagnosis and Treatment of Systemic Lupus Erythematosus, and the Recommendations for the Diagnosis and Management of Lupus Nephritis in China were published. ^[4,5]^ With the goal of promoting the implementation of standard-of-care for patients with SLE, was launched program of COEs in SLE in January 2030. The COEs that could demonstrate high standard in medical service, education and research will be certified. In order to be certified as COEs, the institutions must meet the following requirements.

## Basic Requirements

The COEs must provide high-quality medical service for patients with SLE, offer the state-of-the-art techniques in the diagnosis and treatment of SLE. Additionally, COEs should demonstrate high-level service skills that enabling them to be actively engaged in promoting medical care, teaching, and scientific research in SLE. The COEs should play leading roles and academic progression impact in their regions.

The centers applying for COEs must be affiliated to a tertiary general hospital as a stand-alone department.

## Management of COES

The COEs must provide comprehensive medical services including diagnosis, evaluation, and treatment of SLE as well as the management of comorbidities and complications. The COEs must also have the capability to organize multi-disciplinary consultation if needed.

### Organization

(1) Team members: The medical team of COEs should be composed of rheumatologists with knowledge and experience in the standard-of-care for patients with SLE and led by a dedicated expert in SLE.

(2) Centers: COEs must have outstanding capability in organization and management of patients with SLE, and with a well-established process in triage and patient referral. In addition, each COE must establish a multi-disciplinary team (MDT) that coordinates with interdisciplinary management, consultation and referral. The MDT should include specialties such as nephrology, hematology, pulmonary and critical care, cardiology, neurology, dermatology, *etc*.

### Services

(1) In-hospital services: COEs must have the capacity to promptly admit and provide medical services to newly diagnosed and followed-up patients with SLE. They must have the capability to manage complicated and critical cases and timely admit patients with emergency conditions.

(2) Patient registration: COEs should actively register patients with confirmed diagnosis of SLE using the on-line registry system of SLE.

(3) Equipment and techniques requirement: COEs must have the equipment required for the diagnosis and evaluation of SLE, including imaging for organ involvement evaluation, and techniques such as renal biopsy.

(4) Medications: COEs must have access to the conventional medications of SLE and biological agents.

(5) Follow-up services: COEs must provide for patients with SLE regular and long-term follow-up service.

(6) Patient education: COEs must take the responsibility for patient education, not only focusing on helping patients to better understand the disease, but focusing on improving patient compliance.

### Standard-of-care

(1) The pre-defined number of out-patient visits and in-patient admission must be met at each COE.

(2) COEs should follow the 2020 Chinese Guidelines for the Diagnosis and Treatment of Systemic Lupus Erythematosus for early diagnosis, early intervention and adherent to Treat-to-target (T2T) strategy in order to improve long-term outcomes of patients.

(3) Comprehensive evaluation of disease activity and organ damage should be conducted at a regular base. Individualized treatment regimen should be developed and modified based on the evaluation. Treat-to-target strategy should be followed when prescribing anti-malarial drug, glucocorticoid and immunosuppressive agents.

(4) COEs should focus on prevention and management of comorbidities and complications of SLE. Each COE should have the capability to manage the risk factors and monitor new organ involvement as well as managing severe complications.

(5) COEs should evaluate disease progression, side-effects of medications at a regular base, and stratify patients into different risk groups. Patients at high-risk should be treated aggressively in order to slow organ damage, reduce disease relapse and improve outcomes.

## Requirements for Education and Demonstration of Academic Impact

The COEs should provide training and education to the local rheumatologists as part of the specialists’ training program and continuing medical education program. The purpose of training is to promote the implementation of standard-of-care to clinical practice and improve the overall quality of care. COEs should also demonstrate their academic impact by leading high standard services locally and in neighboring provinces, in order to improve the competency of the local care providers in the region.

(1) Continuing medical education: COEs should have the capability to undertake national continuing medical education programs on standard-of-care for patients with SLE and organize academic conferences in the region.

(2) Physician training: COEs should provide training to local rheumatologists and MDT physicians at a regular base to facilitate the delivery of high standard healthcare services and update the latest guidelines, expert consensus, recommendations and cutting-edge research findings.

(3) Academic impact: COEs should have the capability to organize academic conferences and teaching rounds in city hospitals. They should also have the capability to provide training for visiting rheumatologists from affiliated city hospitals and guide the management for critical cases in the region.

## Requirements For Research

COEs must have high-standard research team and facility. They should also have ongoing research projects in SLE and have the ability in integrating basic science, clinical and translational research. They should at the leading position of research and have academic influence in the region.

(1) Team and facility for research: COEs should have leading investigators in SLE and a high-standard research team to conduct clinical research.

(2) Research program: COEs should actively apply for and participate in national research programs and clinical trials.

(3) Resources for research: COEs should build standardized medical biobank with associated clinical information.

(4) Research network: COEs should be part of the collaborative research network of SLE and have the ability to conduct multi-center and large cohort studies.

(5) Research production: COEs should have high-quality publications of clinical studies in the domestic and international academic journals as the first or corresponding affiliations.

## Structure of COES

(1) The COEs program is initiated by NCRC-DID and funded by the China Health and Medical Development Foundation (CHMDF).

(2) The standing committee appointed by NCRC-DID is responsible for certifying COEs.

## Certification Process

(1) The certification is based on a grading system (Table 1).

**Table 1:** The grading system for Center of Excellence in systemic lupus erythematosus

Classification	Items	Grading Rules	Scores
Basic items (100 points)	Basic requirement (5 points)	The candidate center must be affiliated to a tertiary general hospital with a stand-alone Department of Rheumatology	5 points
	Requirements of organization (20 points)	More than 60% of the department personnels are attending physicians.	5 points
		The department has at least one expert specialized in systemic lupus erythematosus (SLE).	5 points
		◆ Member of national academic associations—5 points	
		◆ Chairman of provincial academic associations—4 points	
		◆ Standing committee member of provincial academic associations—3 points	
		The department has at least two rheumatologists who are experienced in the standard-of-care of SLE.	5 points
		The department has a multi-disciplinary team (MDT) that is composed of experts from nephrology, hematology, respiratory and critical care medicine, cardiology, neurology, dermatology. The commitment of this team is to coordinate with interdisciplinary management, consultation and patient referral.	5 points
	Requirements of service (35 points)	The department registered patients with SLE using the online Chinese Rheumatism Data Center (CRDC) system.	5 points
		◆ A total of 800 or more patients are registered—5 points	
		◆ A total of 500-799 patients are registered—4 points	
		◆ A total of 300-499 patients are registered—3 points	
		◆ A total of less than 300 patients are registered—1 point	
		The department has access to laboratory examinations, including urine test for urine protein quantification, blood tests for serum complement level, serum C-reactive protein level and erythrocyte sedimentation rate.	3 points
		The department has access to serum autoantibody tests, including antinuclear antibody (ANA), anti-dsDNA, anti-Smith, and antiphospholipid antibody profile.	3 points
		The department has the access to imaging examinations, including ultrasound, computed tomography (CT), Magnetic resonance imaging (MRI), *etc*.	4 points
		The department has the access to renal biopsy.	5 points
		The department has the access to conventional medications for SLE and biological agents approved by China Food and Drug Administration (CFDA):	10 points
		◆ Glucocorticoid—2 points	
		◆ Hydroxychloroquine—2 points	
		◆ Immunosuppressants, including mycophenolate mofetil, cyclophosphamide, leflunomide, methotrexate, tacrolimus, cyclosporine and azathioprine—1 point for each but not exceed 4 points;	
		◆ Biological agents, such as belimumab, telitacicept, rituximab, *etc*. —2 points	
		The department provides patient education and follow-up service.	points
	Requirements of stan- dard-of-care (40 points)	The department has more than 300 follow-up patients annually.	15 points
		Over 90% of SLE patients meet the 2012 SLICC or 2019 EULAR/ACR classification criteria of SLE.	5 points
		Over 10% of SLE patients reached lupus low disease activity state (LLDAS) at the last follow-up visit.	10 points
		Over 50% of follow-up patients visit the clinic every six months.	5 points
		Over 80% of follow-up patients are regularly screened for severe complications and organ damages.	5 points
Additional items (50 points)		The department has a standardized medical biobank for sample collection and storage.	10 points
		The quality of autoantibody tests meets the quality requirement of CRDC.	5 points
		The department was certified as clinical research center:	5 points
		◆ National or provincial level—5 points	
		◆ City level—3 points	
		The department was certified as quality control center:	5 points
		◆ National or provincial level—5 points	
		◆ City level—3 points	
		The department provides physician education and training related to SLE:	8 points
		◆ National continuing medical education (CME) program—2 points for each but not exceed 6	
		◆ Routine training for rheumatologists and physicians in MDT—2 points	
		Local academic development and medical training led by the department:	7 points
		◆ Academic conferences and teaching round in city hospitals—1 point for each activity but not exceed 2 points	
		◆ Specialty training for rheumatologists from city hospitals—1 point for each group but not exceed 3 points	
		◆ Remote consultation for critical cases from city hospitals—1 point for each consultation but not exceed 2 points	
		Original articles related to SLE were published or accepted in the last three years (as the first or correspondent affiliation) by the department:	5 points
		◆ JCR Q1—5 points	
		◆ JCR Q2—4 points	
		◆ JCR Q3—3 points	
		◆ JCR Q4—2 points	

JCR, journal citation report.

a) The candidate centers should reach at least 60 points on basic items.

b) Center of Demonstration can only be certified if the candidate centers are scored 60–79 points on basic items and more than 100 points on all items.

c) COEs can only be certified if the candidate centers are scored for more than 80 points on basic items and more than 120 points on all items.

(2). The title of COEs is valid for two years and re-application is needed for renewal.

We believe that the construction of the COEs in the country could remarkably improve the quality of care for patients with SLE in China. We also hope that the developing of the COEs will greatly prompt the standard and level of basic, clinical and translational research for SLE in China. We expect that Chinese patients with SLE will benefit from this COEs development program.
